# Human Eye Optics within a Non-Euclidian Geometrical Approach and Some Implications in Vision Prosthetics Design

**DOI:** 10.3390/biom11020215

**Published:** 2021-02-04

**Authors:** Liviu Bilteanu, Ovidiu I. Geicu, Loredana Stanca, Aurelia M. Pisoschi, Florea Serban, Andreea I. Serban, Valentin Calu

**Affiliations:** 1 Department of Preclinic Sciences, Faculty of Veterinary Medicine, University of Agronomic Sciences and Veterinary Medicine of Bucharest, 105 Blvd. Splaiul Independentei, 050097 Bucharest, Romania; bilteanu.md@gmail.com (L.B.); yo3hfh@yahoo.com (O.I.G.); lory_stanca@yahoo.com (L.S.); aureliamagdalenapisoschi@yahoo.ro (A.M.P.); floreaserban@upcmail.ro (F.S.); 2 Molecular Nanotechnology Laboratory, National Institute for Research and Development in Microtechnologies, 126A, Erou Iancu Nicolae Street, 077190 Bucharest, Romania; 3 Department of Biochemistry and Molecular Biology, Faculty of Biology, University of Bucharest, 91-95 Blvd. Splaiul Independentei, 050095 Bucharest, Romania; 4 Department of General Surgery, University of Medicine and Pharmacy “Carol Davila” Bucharest, 8 Blvd. Eroii Sanitari, 050474 Bucharest, Romania; drcalu@yahoo.com

**Keywords:** human vision, non-Euclidian geometry, artificial visual prosthetic devices

## Abstract

An analogy with our previously published theory on the ionospheric auroral gyroscope provides a new perspective in human eye optics. Based on cone cells’ real distribution, we model the human eye macula as a pseudospherical surface. This allows the rigorous description of the photoreceptor cell densities in the parafoveal zones modeled further by an optimized paving method. The hexagonal photoreceptors’ distribution has been optimally projected on the elliptical pseudosphere, thus designing a prosthetic array counting almost 7000 pixel points. Thanks to the high morphological similarities to a normal human retina, the visual prosthesis performance in camera-free systems might be significantly improved.

## 1. Introduction

Every year, about 1.5 million people worldwide lose their sight as a result of retinal degeneration disorders, among which the most common is retinitis pigmentosa [1], while millions suffer from vision loss due to age-related macular degeneration. Both conditions are characterized by the dysfunction and death of the retinal photoreceptor cells [2], causing irreversible blindness. Photoreceptor cells suffer important damage, while secondary neurons are less affected [2]. In the macula, a significant number of ganglionar cells are still viable at higher eccentricities [3].

Stem cell treatments are an option for medical treatment but they have showed limited success [4], while a promising alternative would be the development of artificial human vision via visual prosthetic devices (VPDs). These devices use reliable intact sensorial processing pathways unaffected by the retinal conditions [5]. Moreover, two reparatory phenomena have been observed in retinitis pigmentosa affected retinas: the amacrine and horizontal cells’ migration into the ganglionar cells layer and neurites growth by the remaining rod, amacrine, and horizontal cells [6]. These changes might enhance VPDs integration and functionality. Though its stepping stones are already set, most VPDs exhibit important issues related to visual acuity, the perception of color and contrast, and vision in low light conditions, which still need solutions.

In terms of image capturing, two VPD types are currently available: the external camera [7,8,9] and the subretinal multiphotodiode array implant [10,11,12,13]. The former VPD class is centered on the use of an external camera fixed on a glasses framework, connected to an epiretinal array consisting of 60 electrodes of 200 µm diameter and a handheld video processor. Argus© systems [8] are the most prominent representative of this class, while other systems working on the same principle are still being tested and improved. The latter class VPDs eliminate the voluminous external camera fixed on the glasses framework, while the implanted stimulation device consists of 1500 photodiodes on a 15 mm × 30 mm surface. The Alpha IMS©, the unique working system of this class, is being tested in Europe, and according to some authors, it is the most promising system in VPDs [14].

Current research evolves via two major approaches: invasive and non-invasive, with the former approach leading to two types of devices—non-retinal and retinal. Retinal VPDs are suitable for epiretinal or subretinal implantation as well as implantation in the suprachoroidal space [15]. Subretinal stimulation has the major benefit of the processing of natural information along the visual path [16]. Visual resolution correlates with the electrode density [14,17], hence a promising way to improve VPDs of the latter type is to increase the number of photodiodes and decrease their distribution surface, aiming to ameliorate some of the above mentioned features (e.g., visual acuity).

In this work, we describe the mathematical foundations of a method to optimize the photodiode arrangement in subretinal implants, and we explain why such an arrangement might improve image capturing and processing.

The hyperbolic geometry framework of human vision provides us with the optimization method. This approach is based primarily on our previous work on the applied gyroscope theory [18] and it is confirmed by the previously reported [19,20,21,22] quantitative data on human retinas obtained by various techniques. Such a framework has an impact on the modeling of human eye function, which can be described by a Klein–Beltrami numerical model generating the macular zones (and also the peri- and inframacular zones). The different photoreceptor cell densities specific to each zone within the human eye were successfully modeled by a system of geodesics providing the positions of the artificial photoreceptor cells (pixels) on an artificial surface. The natural “color sensitivity” of the retinal photoreceptors was modeled by an optimized Fibonnaci color paving of the model surface.

This blend of models of human eye and visual function has an impact on the VPD design and performance. We also discuss some of the challenges of the implementation of a VPD designed according to the model hierarchy presented here.

## 2. Results

### 2.1. Vitreous Humor Charge Vortices Model for Eye Globe Morphology Emergence

The foundation for the model development is the application of a non-Euclidean geometry, i.e., hyperbolic geometry in the description of the human eye morphology and of human vision. Based on our previous work [18], in this section we shall describe the retina, which is the cardinal structure in visual excitation production, using this geometrical formalism.

According to the Anderson-Higgs-Kibble theory [23], the ultra-low frequency oscillations of a terrestrial magnetic field change the water hydrogen bonds (including in the water based biological systems), altering the bonding angles of water dipole molecules and leading to the emergence of a so-called coherence domain.

Extending this theory to the ocular vitreous humor, we postulate that microscopic movement vortices occur within the coherence domain involving the free electrons and positive ions. Through resonance, these microscopic vortices start oscillating with a frequency equal to that of the ultra-low frequency oscillations of the water molecules. Ion species are selected by the local geomagnetic field (B) for specific vortices as a function of their charge/mass ratio [24] through the classical Larmor rotation: $\omega_{i}=q_{i}B/2m_{i}$, where *q_i_* is the algebraic electrical charge and *m_i_* the mass of the moving particle. A permanent geomagnetic energy flux creates many coherent vortices [25], leading to the emergence of macroscopic vortices whose total energy is the sum of the individual microscopic vortices’ energies [24,26]. To these macroscopic vortices we associate, hence group angular velocities which, depending on the moving charges sign, define electron vortices cavities (EVCs) and positive ion vortices cavities (PVCs). Furthermore, gyroscopic cone rotation is determined by the magnetic field [25] generated through the magnetohydrodynamic mechanism [27] by the electric charges movement within the hyaloid channel.

**Figure 1:**
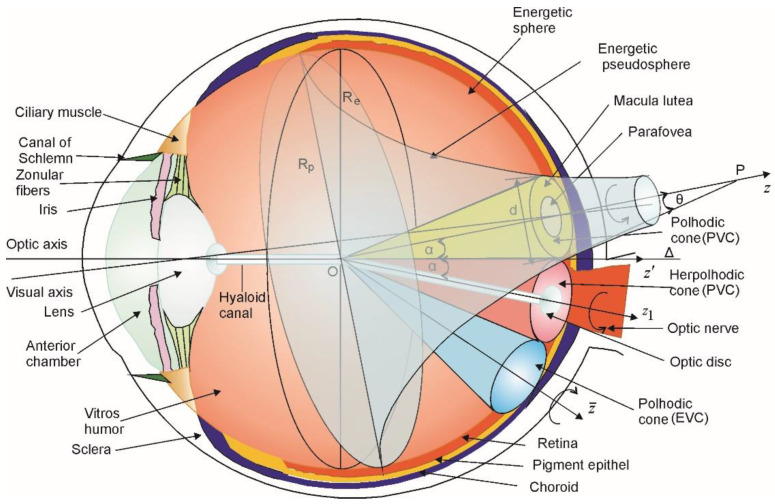
The polhodic and herpolhodic charge vortices are superposed on the main human eye structures represented after [28].

Hence, we apply to the vitreous humor and the retina our previous gyroscope theory conceptually formulated in the terms of non-Euclidian hyperbolic geometric [18]. The following features are illustrated graphically in Figure 1, which are the elements of our hyperbolic geometry approach of human vision.

1. There are three vortices: two polhodic vortices (one associated with PVCs and the other with EVCs) and one herpolhodic vortex associated with a PVC (which will be described below).
  The polhodic vortices have the mobile directions *Oz* and $O\bar{z}$ as symmetry axis, while the herpolhodic one has *Oz_1_* direction along the hyaloid channel as symmetry axis.
  - The PVC polhodic cone (represented in yellow) rotates counter-clockwise with angular speed ***ω*** around the *Oz* axis;
  - The PVC herpolhodic cone (represented in red) rotates counter-clockwise with angular speed ***ω***_1_ around the *Oz_1_* axis;
  - The EVC polhodic cone (represented in blue) rotates clockwise with angular speed ***ω*′** around the axis $O\bar{z}$.
2. The polhodic and herpolhodic vortices are tangent upon a common generatrix Δ, which is in fact the ocular optical axis (Figure 1).
3. The angles between the axis of the herpolhodic ($O\bar{z}$) and polhodic cones (*Oz* și *Oz*_1_) are 2α = 22°44′. The optical axis *Oz′* (Δ) is the bisectrix of the angle between *Oz* and *Oz*_1_.

Within this construction, we shall derive some important geometric features of the macular surface. The kinetic energy of an object submitted only to a multiaxial rotation is given by $E=1/2\sum_{i=1,2,3}J_{i}\omega_{i}^{2}$, where *J_i_* and *ω_i_* are the inertial momentum and the angular velocity with respect to the *“i”* axis. In the energy space, the previous equation defines the Poisson ellipsoid. When *J_i_* = *J* the Poisson ellipsoid is the energetic sphere. Within the $O\bar{\mathrm{xyz}}$ mobile Cartesian system rotating with angular velocity *ω*′ about the $O\bar{z}$, the vector *ω*_1_ is characterized by the Euler angles, $\psi_{e},\phi_{e}\in \left[0,2\pi \right],\theta_{e}\in \left[0,\pi \right]$ defined as: ${\dot{\psi }}_{e}=\omega_{1}$, ${\dot{\phi }}_{e}=\omega \prime$ and ${\dot{\theta }}_{e}=0$, $\theta_{e}=2\alpha$. The last condition is required by the regular precession implied by the existence of coherence domains in the local magnetic field. The index “e” reminds us that the moving particles are electrons. The absolute angular velocity vector Ω_1_ = *ω*_1_+ *ω*′ in the $O\bar{\mathrm{xyz}}$ system has the following components:

$$\{\begin{array}{l} \Omega_{1\bar{x}}=\omega_{1}\sin\theta_{e}\sin\phi_{e}\\ \Omega_{1\bar{y}}=\omega_{1}\sin\theta_{e}\cos\phi_{e}\\ \Omega_{1\bar{z}}=\omega_{1}\cos\theta_{e}+\omega \prime \end{array}$$

These are the parametric equations of a sphere of radius $\omega_{1}$ and centered on (0,0,ω′) in the energy space. This energetic sphere is mirrored in the real space by making a substitution *ω*_1_ → *R_e_*, where *R_e_* = 11.4 mm, thus obtaining the spherical section of the human eyeball. The substitution *ω′ → z_0_* ≠ 0 corresponds to the anatomic shift of 6.3 ± 3.0° of the fovea within the macula with respect to the ocular optical axis.

Symmetrically, within the *Oxyz* mobile Cartesian system rotating with the angular velocity *ω* about the *Oz* axis, the vector *ω*_1_ is characterized by the same Euler angles, *ψ_e_, ϕ_e_, θ_e_* under the same conditions as shown before. Thus, the absolute angular velocity vector Ω_2_= ω_1_ − *ω* in the *Oxyz* has the following components:

(1a) $$\{\begin{array}{l} \Omega_{2x}=\omega_{1}\sin\theta_{e}\sin\phi_{e}\\ \Omega_{2y}=\omega_{1}\sin\theta_{e}\cos\phi_{e}\\ \Omega_{2z}=\omega_{1}\cos\theta_{e}-\omega \end{array}$$

Performing the substitution: *ω* = −*ω*_1_ ln [tan (*θ_e_*/2)] one obtains the equation of a pseudosphere of negative curvature consequently called negative energy pseudosphere, with the following components:

(1b) $$\;\{\begin{array}{l} \Omega_{2x}=\omega_{1}\sin\theta_{e}\sin\phi_{e}\\ \Omega_{2y}=\omega_{1}\sin\theta_{e}\cos\phi_{e}\\ \Omega_{2z}=\omega_{1}\left(\cos\theta_{e}+\ln\left[\tan\left(\theta_{e}/2\right)\right]\;\right) \end{array}$$

which is mirrored from the energy space into the real space by a scaling substitution *ω*_1_ → *R_p_*. In this way, we obtain the following surface:

(2) $$\{\begin{array}{l} X=R_{p}\sin\theta_{e}\sin\phi_{e}\\ Y=R_{p}\sin\theta_{e}\cos\phi_{e}\\ Z=R_{p}\left(\cos\theta_{e}+\ln\left[\tan\left(\theta_{e}/2\right)\right]\;\right) \end{array}$$

Thus, in our case, the motion of the vector Ω_1_= *ω*_1_ + *ω′* generates an energetic sphere of algebraic positive energy of radius *R_e_*, represented in light red, and the motion of the vector Ω_2_ = *ω*_1_ − *ω* generates an energetic pseudosphere of radius *R_p_*, represented in light blue [18]. Otherwise said, the energetic sphere is generated by the movement of the negatively charged particles (electrons), while the energetic pseudosphere is generated by the movement of the positively charged particles.

Moreover, the intersection of the energetic sphere and pseudosphere defines the macular surface. The average measured macular diameter *d* = 5.5 mm [29] is obtained for *R_e_* = 11.4 mm and it corresponds to an angle of 2α [18].

### 2.2. Macula Representation as an Elliptical Pseudosphere

In the previous section, we have described the macular zone as a pseudospherical sphere section which corresponds, according to the measurements, to a solid angle of 0.34°–18.20° (0.35–5.5 mm diameter) [30]. The macular morphology as a negative curvature surface [31], which is different from the ideal pseudospherical surface, emerges as the result of the interaction between the energetic sphere and pseudosphere within the wide context of the mechanical forces field acting on the biological tissue during embryological development. The meridian of such a negative curvature revolution surface is given by [32]:

(3) $$Y=\int^{\;}\sqrt{\frac{R_{p}^{2}-X^{2}}{X^{2}+R_{e}^{2}-R_{p}^{2}}}dX$$

**Figure 2:**
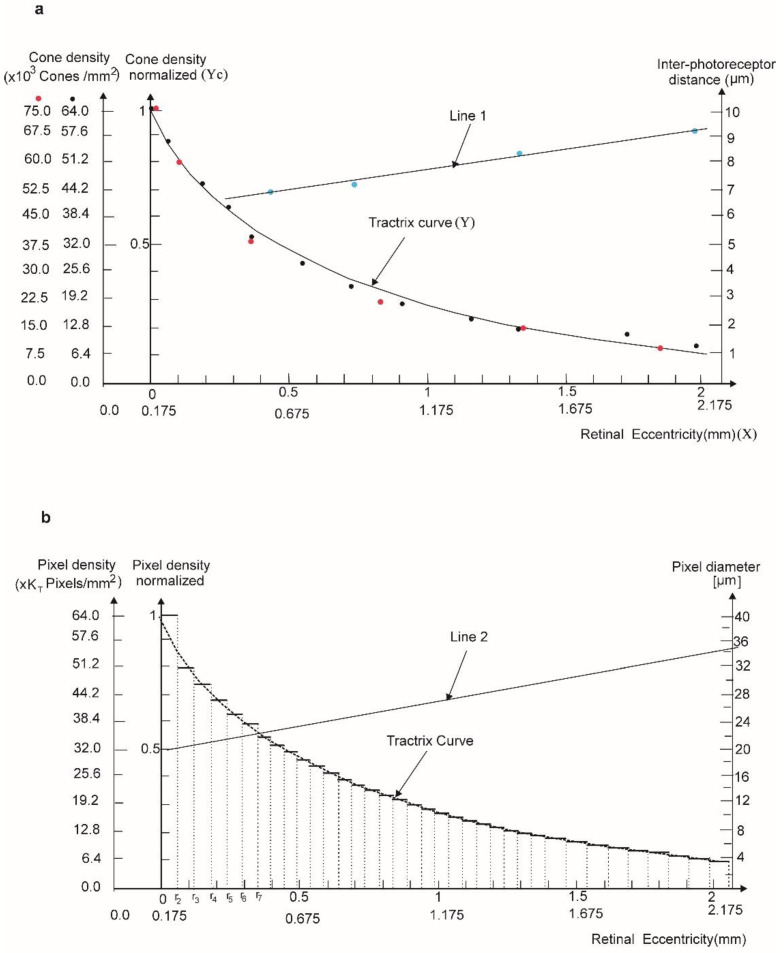
The shape and characteristics of cone density surface in macula. (**a**) Normalized cone density (black points are data from [20] and red points are data from [21] and the tractrix fitting curve. Line 1 is the interpolation of the inter-photoreceptor cell distances as a function of eccentricities data set in [20]. (**b**) The pixels’ eccentricity distribution on the artificial human macula (AHM) in elliptical shape achieved by an arrangement of photoreceptors of 20 μm. Line 2, parallel to line 1, shows the linear pixel diameter increase with increasing eccentricity.

Such a curve can be used to model the cone cell distribution in the macular region. Indeed, recent precise measurements on cone cell distribution are available in the literature and they are represented in Figure 2a (black points are data from [20] and red points are data from [21]). Actually, the data points (*Y_c_*) plotted in Figure 2a are obtained by the normalization of the actual cell density to the maximal cone density (64 × 10^3^ cones/mm^2^ for the densities reported by [20] and 75 × 10^3^ cones/mm^2^ for the densities reported by [21]). The normalization is suggested by the different measurement methods employed in these references. 

The fitting curve equation in Figure 2a is derived from Equation (3) for *R_p_* = *R_e_* = *R* and then translated by −*r*, i.e., *Y* → *Y* + (−*r*), that is:

(4) $$Y=-r+R\left(\ln\frac{R+\sqrt{R^{2}-X^{2}}}{X}-\frac{\sqrt{R^{2}-X^{2}}}{R}\right)$$

For *r* = 0.175 mm and *R* = 2.75 mm, the fitting of experimental data (*Y_c_*) in Figure 2a satisfies the Tschebyshev inequality max |*Y_c_* − *Y*| ≤ 3*D* = 6.42, whereas *D* = 2.14 is the standard deviation and shows low deviation of measurement data points from tractrix form (4). 

In order to represent the tractrix in the variables *R* and *θ* in which the approach was formulated in the previous section, in Equation (4) we perform the following substitution:

(5) $$\cos\theta =-\frac{\sqrt{R^{2}-X^{2}}}{R}$$

By simple trigonometric calculations, one obtains:

(6) $$\tan\frac{\theta }{2}=\frac{R+\sqrt{R^{2}-X^{2}}}{X}$$

Such that Equation (4) becomes:

(7) $$Y=-r+R\left[\ln\left(\tan\frac{\theta }{2}\right)+\cos\theta \right]$$

**Figure 3:**
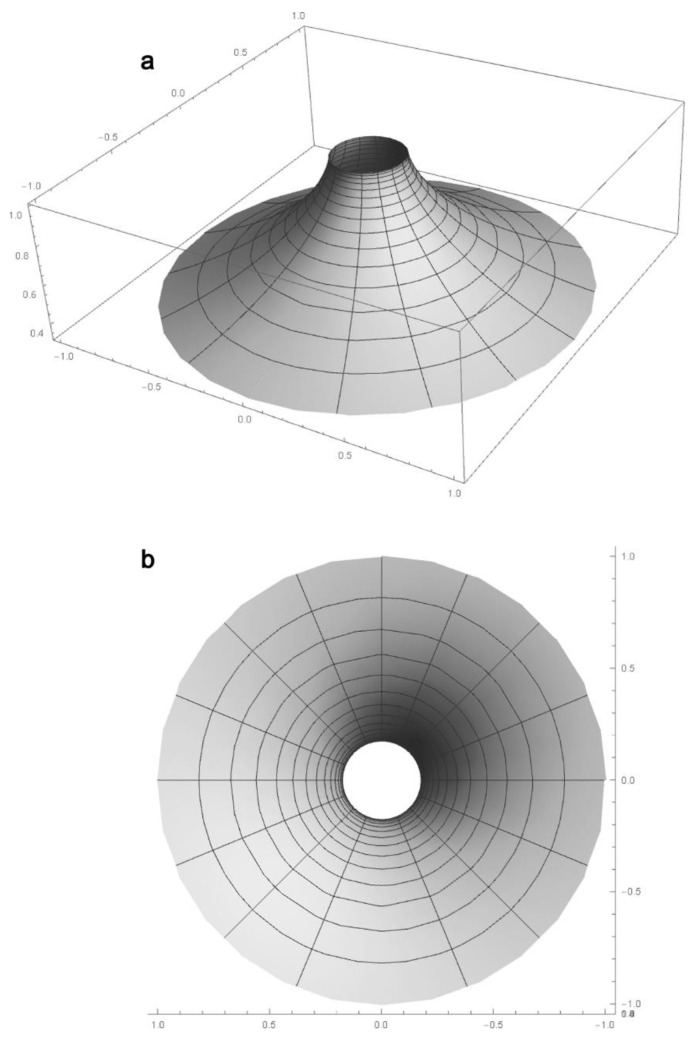
Elliptic pseudosphere generated by Equation (6) with *r* = 0.175 mm and *R* = 2.75 mm as the geometrical representation of the human macula: lateral view (**a**) and axial view (**b**). The graph has been realized using Wolfram Mathematica 10.

The macular numerical representation is obtained by rotating the curve (7) around the visual axis, leading to the creation of an elliptical pseudosphere with the following parametrical equations:

(8) $$\{\begin{array}{l} x=\left\{-r+R\left[\cos\theta +\mathrm{lntan}\left(\theta /2\right)\right]\right\}\sin\varphi \\ y=\left\{-r+R\left[\cos\theta +\mathrm{lntan}\left(\theta /2\right)\right]\right\}\cos\varphi \\ z=R\sin\theta \end{array}$$

where 0 < *θ* ≤ *π*/2, 0 ≤ *φ* ≤ 2*π* (Figure 3). Through its rotation, around the cone density axis (visual axis), the tractrix generates a cylinder of radius 0.175 mm and also an elliptic pseudosphere surface (EPS) [33], suggesting that the macular cones are distributed on a hyperbolic surface, thus the hyperbolic geometrical features of the human visual function (Figure 3).

We state here the first quadratic differential form for the surface described by EPS:

(9) $$\varphi_{1}=ds^{2}=Ed\theta^{2}+2Fd\theta d\varphi +Gd\varphi^{2}$$

where

(10) $$\{\begin{array}{l} E={\left(\frac{\partial x}{\partial \theta }\right)}^{2}+{\left(\frac{\partial y}{\partial \theta }\right)}^{2}+{\left(\frac{\partial z}{\partial \theta }\right)}^{2}=R^{2}\cot^{2}\theta \\ F=\frac{\partial x}{\partial \theta }\frac{\partial x}{\partial \varphi }+\frac{\partial y}{\partial \theta }\frac{\partial y}{\partial \varphi }+\frac{\partial z}{\partial \theta }\frac{\partial z}{\partial \varphi }=0\\ G={\left(\frac{\partial x}{\partial \varphi }\right)}^{2}+{\left(\frac{\partial y}{\partial \varphi }\right)}^{2}+{\left(\frac{\partial z}{\partial \varphi }\right)}^{2}=R^{2}T^{2}\\ T=\left(-r/R+\cos\theta +\mathrm{lntan}\left(\theta /2\right)\right)<0 \end{array}$$

while the second quadratic form defined:

(11) $$\varphi_{2}=Ld\theta^{2}+2Md\theta d\varphi +Nd\varphi^{2}$$

has the coefficients given by the formulas:

(12a) $$L=\frac{1}{\sqrt{EG-F^{2}}}\left|\begin{array}{lll} \frac{\partial^{2}x}{\partial \theta^{2}} & \frac{\partial^{2}y}{\partial \theta^{2}} & \frac{\partial^{2}z}{\partial \theta^{2}}\\ \frac{\partial x}{\partial \theta } & \frac{\partial y}{\partial \theta } & \frac{\partial z}{\partial \theta }\\ \frac{\partial x}{\partial \varphi } & \frac{\partial y}{\partial \varphi } & \frac{\partial z}{\partial \varphi } \end{array}\right|=-R\cot\theta$$

(12b) $$M=\frac{1}{\sqrt{EG-F^{2}}}\left|\begin{array}{lll} \frac{\partial^{2}x}{\partial \theta \partial \varphi } & \frac{\partial^{2}y}{\partial \theta \partial \varphi } & \frac{\partial^{2}z}{\partial \theta \partial \varphi }\\ \frac{\partial x}{\partial \theta } & \frac{\partial y}{\partial \theta } & \frac{\partial z}{\partial \theta }\\ \frac{\partial x}{\partial \varphi } & \frac{\partial y}{\partial \varphi } & \frac{\partial z}{\partial \varphi } \end{array}\right|=0$$

(12c) $$N=\frac{1}{\sqrt{EG-F^{2}}}\left|\begin{array}{lll} \frac{\partial^{2}x}{\partial \varphi^{2}} & \frac{\partial^{2}y}{\partial \varphi^{2}} & \frac{\partial^{2}z}{\partial \varphi^{2}}\\ \frac{\partial x}{\partial \theta } & \frac{\partial y}{\partial \theta } & \frac{\partial z}{\partial \theta }\\ \frac{\partial x}{\partial \varphi } & \frac{\partial y}{\partial \varphi } & \frac{\partial z}{\partial \varphi } \end{array}\right|=-RT\sin\theta$$

The total (Gaussian) curvature of the EPS is:

(13) $$K=\frac{LN-M^{2}}{EG-F^{2}}=\frac{1}{R^{2}}\frac{\tan\theta \sin\theta }{T}<0$$

The equation that defines the geodesic lines of a surface [32] is:

(14a) $$\frac{d\varphi }{d\theta }=\pm \sqrt{\frac{E}{G}}\tan\alpha_{g}$$

Or

(14b) $$\alpha_{g}=\pm \arctan\left(\frac{d\varphi }{d\theta }\sqrt{\frac{G}{E}}\right)$$

when the tangent to the geodesic curvature forms angle *α_g_* with the tangent to the line of the orthogonal coordinates system. If the expressions for *E* and *G* mentioned above are introduced in the system of Equation (14a,b) and then they are integrated, we obtain the geodesics family for the EPS as follows:

(15) $$\{\begin{array}{l} \varphi =\pm \tan\alpha_{g}\int^{\;}\frac{\cot\theta }{-r/R+\cos\theta +\mathrm{lntan}\left(\theta /2\right)}+\varphi_{0}\\ \sin\alpha_{g}\sin\theta =c_{0} \end{array}$$

where *φ*_0_ and *c*_0_ are integration constants. The second equation of system (15) represents a constant associated with a given geodesic and it is called the first integral of the geodesic.

Until now, we have explored the case *R_e_ ≈ R_p_*, leading to the energetic pseudospherical surface specific to humans and primates. However, if *R_e_ < R_p_* the macular surface has a typical elliptic pseudospherical shape with negative curvature exhibiting a sharp edged fovea (as in birds, in some reptiles and in fish species), and finally, if *R_e_ > R_p_* (as in raptor vision) the macular surface has a hyperbolic pseudospherical shape exhibiting a supplemental fovea [33]. All these features being obtained from the generatrix Equation (3) suggests the importance of hyperbolic geometry in analyzing the visual function, in general, and the macular retina, in particular.

### 2.3. Macula Zones Generation within Klein–Beltrami Model 

In this section, we shall derive the plane projection of the above macular surface by generating it through a succession of elementary operations performed on hyperbolic geometry triangles. We shall prove that specific retinal zones (i.e., macula, fovea, fovea avascular zone and umbo) can be rigorously parameterized within an elementary hyperbolic geometry HG geometrical modeling in excellent agreement with the measurement data [30] (see Table 1). 

**Figure 4:**
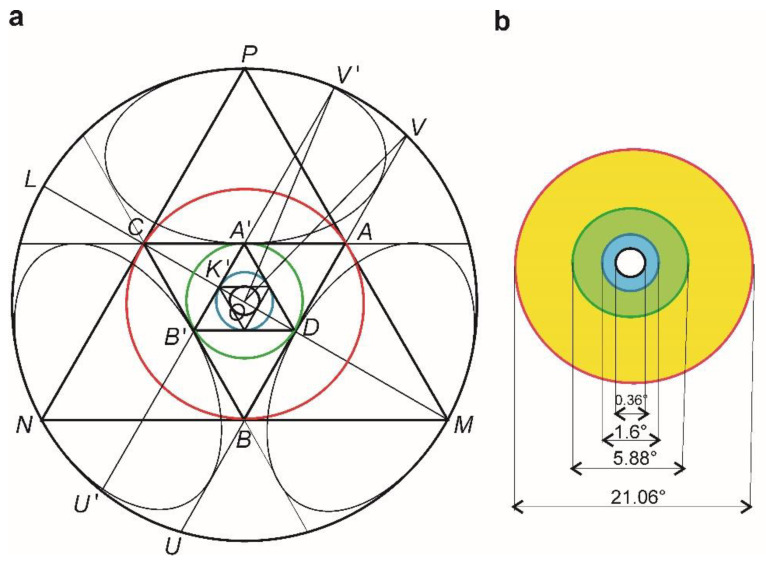
The macula hyperbolic structure. (**a**) In the HGKB model, the equilateral triangles (EQTs) successively inscribed to the horizon circle lead to a correspondence between the hyperbolic angles of the triangles complementary to 60° and the angular eccentricities of the macular regions. The red circle represents the macula, the green circle represents the fovea (FV), and the blue circle represents the foveal avascular zone (FAZ), while the black circle is the umbo. (**b**) The yellow disc comprises the perifoveal and parafoveal areas, the green one represents the FV and the blue disc represents the FAZ and foveal areas, while the white disc represents the umbo.

We start with a reminder that within HG, a triangle is fully and uniquely described if either all its angles or all its side lengths are known, thus the existence of similar triangles (such as in Euclidian geometry) is excluded. If an equilateral triangle (EQT) is inscribed *MNP* (see Figure 4a) in a unit circle (*R* = 1) (according to the hyperbolic geometry Klein–Beltrami (HGKB) model [34]), then its sides are infinite in length and its angles are equal to zero. 

**Figure 5:**
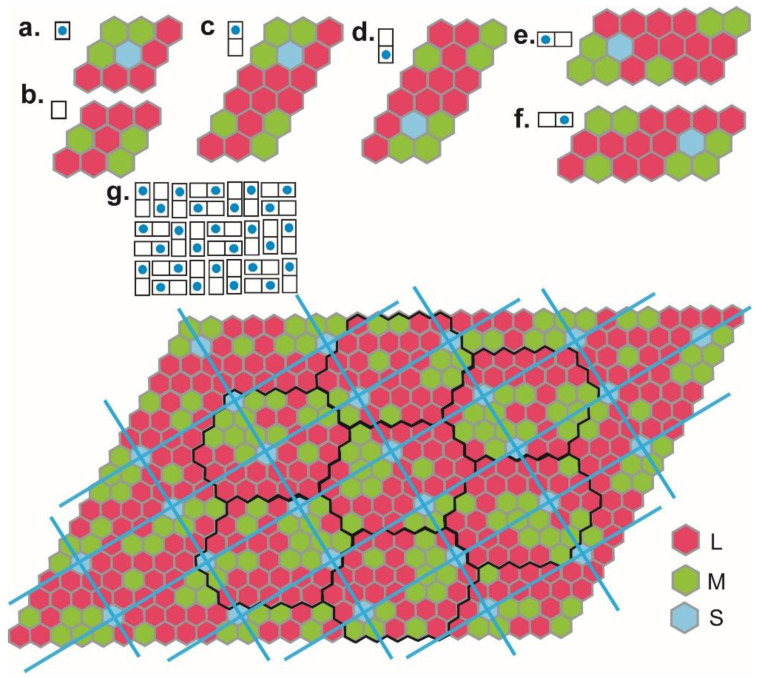
The 2D visual mosaic. (**a**) The basic structural element. (**b**) The supplementary structural element. (**c**) A direct orientation panel. (**d**) An inverted orientation panel. (**e**,**f**) horizontal replicas of the direct and inverted orientation panels. (**g**) A 2 × 3 panel [42] obtained using the direct paths and all the permutations. In the upper left, the tiling combination algorithms are given. Short (S), medium (M) and long (L) cone cell sensitivity is represented by the corresponding colors: red (620–750 nm), green (500–570 nm) and blue (450–500 nm), respectively.

In the *MNP* triangle, we can inscribe another EQT *ABC*, with its side lengths equal to 1.92 and its angles equal to 38.94°, within which we shall inscribe another EQT with 0.887 side length and its angles equal to 54.12° (Figure 5a). By successively inscribing triangles within triangles, we observe that their side lengths decrease and their respective angles converge increasingly towards the value of 60°, without ever reaching this value, because in HG the sum of triangle angles is smaller than 180°. This observation offers insight about how far away the triangle is from the horizon circle or the circle’s center. Thus, we obtain a set of concentric annular areas which are a rigorous geometrical model (Figure 4b) for the macular region and the surrounding infra and perimacular zones, whose diameters are close to the measured values (see the proof below).

Next, we shall use the HGKB model to support the retina hyperbolic features. Let *M*, *N* and *P* be three ideal points. Points *A*, *B*, *C* are added such that *M*, *N* and *P* are the centers of the holocircles exinscribed to ∆*ABC*. The triangle ∆*ABC* is an EQT because ∆*MNP* has infinite quota, and it is an ideal EQT as its three hyperbolic angles are null (Figure 4a).

Since the center of the horizon circle is the same as that of the circle circumscribed to ∆*MNP* and as that of the circle inscribed within ∆*MNP*, we use Euclidean geometry for the EQT. All the terms are proportional with the horizon circle radius *R = OV* considered equal to 1. We have *NP* = *PM* = *MN* = $\sqrt{3}$ and thus: *AB* = $\sqrt{3}$/2. Furthermore, in the right-angle triangle *DOV*, we have: *OD* = 1/4, *OV* = 1, therefore *DV*^2^ = *OV*^2^ − *OD*^2^ = $\sqrt{15}$/ 4. 

Hence *AV* = *DV* − *DA* = $\left(\sqrt{15}-\sqrt{3}\right)/4$. Since *BU* = *AV*, we have *AU* = *BU* + *AB* = $\left(\sqrt{15}+\sqrt{3}\right)$/4. We introduce the following notation *ρ_h_* = *AU/AV* called the anharmonic ratio of the points *A*, *B*, *U* and *V*. When calculating this ratio, one obtains *ρ_h_* =$\left(\sqrt{15}-\sqrt{3}\right)/\left(\sqrt{15}+\sqrt{3}\right)$ = Φ^2^, where Φ^2^ is the golden ratio within the first EQT. The hyperbolic distance between *A* and *B* is given by $d\left(AB\right)=2{\ln\Phi }^{2}=4\ln\Phi$ from which we obtain *AB = BC = CA =* 1.92 [34]

Now let us calculate the angles. The angle *A* can be computed using the formula [35]:

(16) $$\cos∡A=\frac{ch\left(a/R\right)}{1+ch\left(a/R\right)}$$

where *R =* 1 and *a* is the EQT side. Hence, cos∡A =(ch(4lnΦ))/((1+ch(4lnΦ))=0.7778 or $∡A=38.94^{{}^{\circ}}$, a value close to one known from Fibonacci’s geometry. 

In the circle inscribed to ∆ABC, we can inscribe another EQT, *A′B′D*, where *OV′* = 1 is the radius of the inscribed circle and *OK′* = 1/8; then from the right angle triangle *OK′V′* we can obtain *K′V′*^2^ = *OV′*^2^ − *OK′*^2^ and *K′V′* = $\sqrt{1-\left(1/64\right)}$ = $\sqrt{63}/8$. However, *A′V′ = K′V′ − K′A′* or *A′V′* = $\left(\sqrt{63}-\sqrt{3}\right)$/8 = $\sqrt{3}\left(\sqrt{21}-1\right)$/8. In order to calculate *ρ′_h_ = A′U′/A′V′* the anharmonic ratio of points *U′*, *B′*, *A′*, *V′*, we shall first calculate the length *U′A′*, where *U′B′ = A′V′* as follows *U′A′* = *U′B′* + *B′A′* = *A′V′* + *B′A′* = $\sqrt{3}\left(\sqrt{21}+1\right)$/8. Thus, the ratio *ρ′_h_ =*$\left(\sqrt{21}+1\right)$/ $\left(\sqrt{21}-1\right)$ = 1.558, allowing us to calculate the hyperbolic distance d(*A′B′*) = 2 ln (1.558) = 0.887136. Likewise, we are able to calculate the angles of the EQT *A′B′K′* as cos(∡A′) = [ch d(*A′B′*)/[1 + ch d(*A′B′*)] = 0.586, and finally we obtain the value of 54.12° for the angle *A′*.

We can proceed in a similar manner by successively inscribing additional EQTs with smaller and smaller sides, while their angles increase slightly towards the value of 60º—however, they never reach it. Thus, for the next inscription, following every two terms of the square root of Fibonacci numbers 5, 21, 89 and 377, we obtain *ρ″_h_ = A″U″/A″V″* the anharmonic ratio of points *U″*, *B″*, *A″*, *V″*, *ρ″_h_ =*$\left(\sqrt{89}+1\right)$/ $\left(\sqrt{89}-1\right)$ = 1.237. Thus, d(*A″B″*) = 2 ln (1.237) = 0.425 and cos(∡A″) d(*A″B″*)/[1 + ch d(*A″B″*)] = 0.522, or thus ∡A″= 58.40°. Further, for the Fibonacci number 377 we have $\rho {\prime \prime \prime }_{h}=1.1086$ and the calculations are performed in the same manner.

The EQT circumscribed circles, inscribed one within the other, are in a direct relationship with the circles which define the macular areas. The complementary to 60º of the *A, A′, A″, A‴* angles denoted by *A_c_, A_c_′, A_c_″, A_c_‴*, respectively, is in correspondence to the measured eccentricity angles of the macular regions (Table 1).

There is an obvious correspondence between the macular structure in HG and Euclidean geometry representations in [30,36] (Figure 4b). The fovea FV area is approximately the same size in all species of monkeys and humans, although they have large differences in retinal surfaces, supporting the idea that physiological constraints induce the approximate size of the FV [37].

### 2.4. Discrete Model for Artificial Photo-Receptor Cell Distribution

#### 2.4.1. Photoreceptor Cell Zones on Artificial Human Macula Surface

The human retina surface contains two types of sensory cells: about 7 × 10^6^ cone cells and 1.25 × 10^8^ rod cells, for color vision and low light vision, respectively, which are sensitive to light with wavelengths between 400–750 nm. 

When seeking to imitate the natural structural features of the retina, this distribution dictates the localization of eventual artificial photoreceptor cells (from now on called throughout this text “pixels” or “photodiodes”) on a prosthetic surface. In the light of our numerical model of the macular surface, this is achieved by using pixels of various dimensions distributed on the elliptic pseudosphere with a diameter of 5.5 mm (Figure 3), which actually represents an artificial human macula (AHM). The AHM should be surgically implanted in the macula between the pigment epithelium and the bipolar cells—its main role being to convert the incident light into pulsed photocurrent. 

Several reasons supported our choice for hexagonal shaped photodiodes. As we mentioned in the text, the hexagonal tiling of cone photoreceptors arranged in the annular surface in the FV [21] provides an optimal solution for generating the necessary signals after proper photoexcitation, as proved in previous works [38]. It has been shown that hexagonal shaped photodiodes have a higher coverage factor and higher angular resolution [39]. Beyond these optimized physical parameters, it has been shown before that hexagonal based configurations use less storage and computations in signal processing [38]. Finally, but not less important, within the optimization framework of our work, there is a formal reason for the choice of hexagonal shapes: a Veronoi analysis correlated on the spatial frequency of cone cells as shown in [21]. Moreover, in the human retina, photoreceptor diameters at FV are between 1.6 and 2.2 μm [40] and the inter-photoreceptor distances are between 6.8 and 9.3 μm—both of them increased with the retinal eccentricities [21]. Although the cones pave a curved surface, the distance between them and the diameter exhibit a linear increase (line 1, Figure 2a) with the retinal eccentricities [21]. In the fovea, they are separated by distances of 28 μm, while in the rest of the macula these separation distances progressively increase up to 2 times greater than the pixel dimension (40 μm) [40] 

In order to imitate such an important feature, in view of the findings in the previous section, the AHM is mapped by a succession of concentric annular hyperbolic zones (AHZs), within which the photoreceptor diameter and inter-photoreceptor distances are quasiconstant. The AHM should be generated thus by the discrete tractrix (Figure 2b) derived from the continuous tractrix curve in Figure 2a, depending on the pixel dimension and on the pixel number in the discretization range. 

The discrete generating tractrix is thus dependent on the pixel surface density defined through a storage density coefficient *K_T_*, which is the ratio between the pixel density (number of pixels/mm^2^) in the annular hyperbolic zone (AHZ) and the observed value on the “natural pixel” (i.e., cone) density. The value of $K_{T}$ defined as a superficial cell density is a spatial distribution parameter related to the cone cell arrangement in a normal human macula. As mentioned before, the macula can be described in a 2D projection as a succession of annular concentric regions with different cone cell distributions or densities. Due to cone cell dimensions, natural variation with the eccentricity, i.e., position with respect to the geometrical center of the human macula $K_{T}$ has an approximate constant value of 1000 for each of these regions. More precisely, $K_{T}$ is eccentricity independent while cone cell diameters are eccentricity dependent. Within an ideal prosthesis perfectly imitating such a configuration, $K_{T}$ should be as close as possible to 1000, while photodiodes replacing the cone cells should modify their dimensions quasi-continuously according to their position (eccentricity) on an elliptic pseudosphere shaped macular prosthesis. The major technological challenge is the ability to build such a device with constant density coefficient $K_{T}$ and a photodiode diameter versus eccentricity distribution obeying the linear relation represented by the Line 2 in Figure 2b. Any major deviations from these specifications will lead to visual perception distortions. Today, technological advances allow the development of pixels with higher diameters, leading to two orders of magnitude differences between the natural cone density of the human eye and the corresponding artificial density of the order of tens of pixels per mm^2^ (*K_T_* = 1000).

If one considers a pixel diameter of 20 μm in the first AHZ, whose width is 3 pixels, we have *K_T_* = 32 (Figure 2b). The entire AHM surface is then covered by pixels of linearly increasing diameters as a function of the retinal eccentricities. The line 2 in Figure 2 shows the linear pixel diameter increase with increasing eccentricity plotted as a parallel to the line number 1 (in Figure 2a). This is the mathematical expression of the prosthetic features closely related to the natural retina feature. In this way, within an AHZ one can place 162 pixels with a required diameter satisfying (in pixel density axis/mm^2^) the measured density corresponding to the average radius as it shown in (a).

#### 2.4.2. Fibonacci Mathematical Model for Color Pixel Optimized Paving 

Having obtained the pixel densities in relation to their respective linearly varying diameters, it is necessary to establish the pixel color spatial distribution in order to obtain features similar to those in the natural macula. To achieve this, we consider, as a ground hypothesis, that a cone cell distribution pattern be obtained by a “construct method” [41]. In the framework of this method, in order to describe a macroscopic object exhibiting a certain symmetry feature, that object is divided into a finite set of basic structural elements, which have, at small scale, the same structural features as the macroscopic object. In our case, the macroscopic object is the cone cell distribution itself, our task being hence to find the basic structural elements that should exhibit the same ratio of cone cell types. 

**Figure 6:**
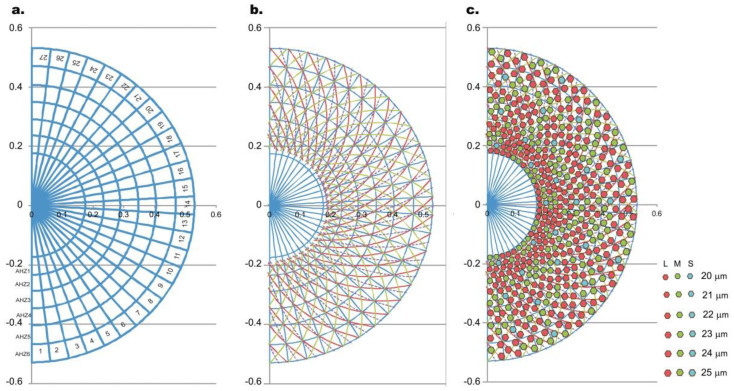
Packing arrangement of color pixels for 2 × 3 panel board (**a**) The lattice obtained by the planar projection of the superposition of the 27 circular surfaces on the first 6 annular hyperbolic zones (AHZs) resulting in a configuration of 162 circular trapezoids. (**b**) Three types of geodesics obeying Equation (15) have been plotted on the circular trapezoid configurations: the continuous red and green lines (*c*_0_ = 0.223) passing through the trapezoidal corners (one pixel lies on each line) and the dotted purple line (*c*_0_ = 0.127) on which lies the third pixel inside the trapezoid as explained in the text. (**c**) The final projection of 2 × 3 panels into various AHZs. This plot has been obtained in Microsoft Excel 2010 by a routine implementing the method described in the text. S, M and L cone cell sensitivity is represented by the corresponding colors: red (620–750 nm), green (500–570 nm) and blue (450–500 nm), respectively.

**Figure 7:**
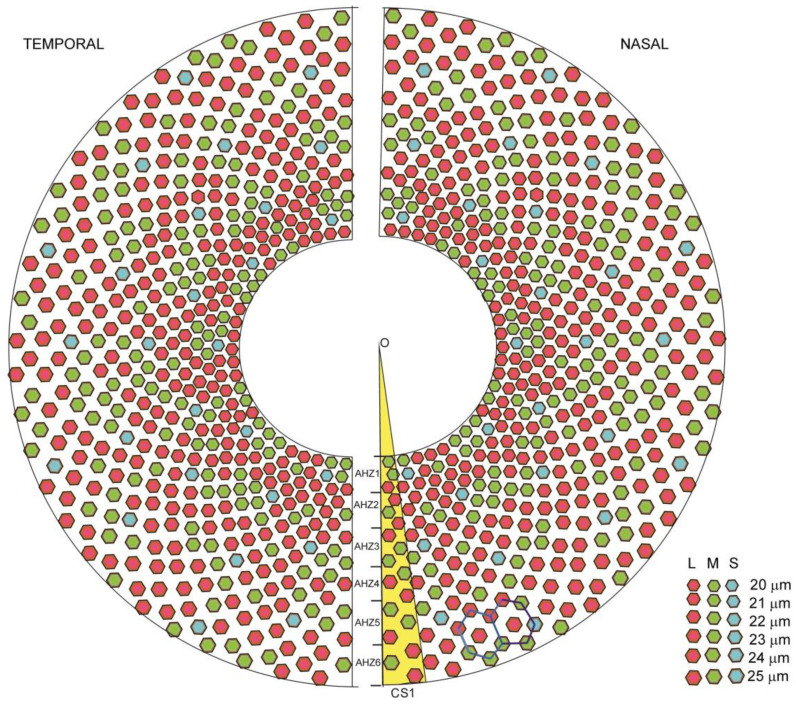
The organization pixel mosaic in FAZ. The FAZ zone in the artificial human macula (AHM) is composed by two hemipseudospheres first, the hemipseudosphere generated like in Figure 6 represents the nasal part of the retina and the other hemipseudosphere is the mirror imagine of the first one, being the temporal part of the retina. Each hemipseudosphere contains 486 pixels in 2 × 3 panels such that the each half pseudosphere contains 486 pixels organized in 2 × 3 panels, such that the entire prosthesis hosts 972 pixels. Each two adjacent circular trapezes determines a hexagonal arrangement of pixels marked by hexagons with gray lines. The first circular surface is marked in yellow. This plot has been obtained in Microsoft Excel 2010 by a routine implementing the method described in the text. S, M and L cone cell sensitivity is represented by the corresponding colors: red (620–750 nm), green (500–570 nm) and blue (450–500 nm), respectively.

The cone cells exhibit higher density in the central region of the retina, more exactly within an area of 5.5 mm in diameter [36] of the macula. The cone cells are sensitive to short (S), medium (M) and long (L) wavelength regions within this visible electromagnetic spectrum, being called accordingly S, M and L cells [43]. These regions are: blue (450–500 nm), green (500–570 nm) and red (620–750 nm), according to the color they represent. The correspondence between color and cone cell sensitivity is used in Figure 5, Figure 6 and Figure 7 to represent the configurations of artificial photosensitive cells (i.e., pixels or photodiodes).

We state the retinal structure is the following [44]: 11.11% S type cells, 33.33% M type cells and 55.56% L type cells. Roughly, the retinal structure cell ratio is 1S:3M:5L, hence each basic structural element able to perceive the complete visible spectrum would be composed of nine or eighteen cone cells in the same ratio (Figure 5a–f). To reach the proportions specific to the human retina, it is necessary to add a supplementary structural element, comprised of nine cells, with the ratio 3M:6L (Figure 5b). Together, basic and supplementary structural elements contain 18 cone cells (Figure 5c–f), with the frequency ratio 1S:6M:11L, corresponding to the proportions of 5.5% S type, 33.33% M type and 61.11% L type matching those described by retinal morphology [45]. 

Morphological studies have revealed the mean proportion of the cone cell types (all at 1° eccentricity) to be: L 62.97% (±3.396%), M 31.31% (±3.396%) and S 5.72% (±0.69%) [45]. It is currently considered that in the FV central region, the M and L cone cell type distribution appears to be a nonrandom structure in some individuals [46], while the S type is more regular than would be expected randomly [45,47], being completely absent in the very center of the FV in a region of about 0.34°. The latter feature makes the FV an area of maximal visual acuity enhanced by the macular elliptical isodensity contour [40]. 

Pixel color distribution was optimized in a Fibonacci sense using the domino tiling method [42]. An 18-cone cell unit (one basic and one supplementary structural element) can be arranged within one 2 × 1 panel, with a direct orientation (Figure 5c), and its inverted image is a 2 × 1 panel (Figure 5d). The horizontal 2 × 1 panel is shown in Figure 5e and its inverted image is shown in Figure 5f. Using the 2 × 1 panel, a larger 2 × 3 panel with all the possible combinations can be built (Figure 5g). Although the algorithm used to obtain these patterns is very precise, the red and green cone cell types seem to be distributed randomly, and only the blue ones maintain certain symmetry. The blue segments in the 2 × 3 panel represent the geodesics of the EPS onto which the S-type cones are arranged. The hexagons [48] are marked with black lines (a simple hexagon has 36 cone cells and a composed one has 252 cones). The total number of cone cells for a 2 × 3 panel is 486 (Figure 5g). This distribution of cone cell types in the normal retina is supported by previous reports suggesting that L versus M gene expression is mediated by a random genetic process [49]. The S type cells are located at the cross points of EPS geodesics (Figure 5g). This explains why changes in illumination influence the hyperbolic distances and the geodesics [50]. 

#### 2.4.3. Photoreceptor Cell Positions in the Annular Hyperbolic Zones

The implementation of the circular shape of the color arrangement in the previous section implies that each column in the arrangement in Figure 5g (for example) turns into a circular surface. Therefore, in each circular trapezoid or sector it is possible to position three hexagonal pixels with the corresponding colors as shown in Figure 5g, which together with the two circular sectors forms a hexagonal arrangement of the pixels (Figure 6). 

In order to describe rigorously how we apply the 2D model in the previous section to a 3D pseudospherical surface, we introduce the annular hyperbolic zones (AHZs), which are a tool to discretize the prosthetic surface into regions with different pixel dimensions but homogenous pixel distribution. This is the point when color distribution in Euclidean planar coordinates is transformed into non-Euclidian (hyperbolic) coordinates. 

For example, for the initial radius *r*_1_ of 0.175 mm, the circumference of the smallest circle of the pseudospherical surface is 1.099 mm. Within this surface, respecting the previous color distribution model, one can position 54 pixels of 20 μm diameter. Thus, the projection of the pseudospherical macula on the plane can be divided into 54 circular sectors, and each sector corresponds to a circular angle of 360/54 = 6.66°. The intersection of these sectors with the 3 pixel-wide AHZ (which in the plan become circular areas) defines some circular trapezoidal surfaces. To achieve optimal light processing, on each of these surfaces the 3 pixels can be arranged in non-collinear configurations.

Thus, an AHZ has its circular limits defined by two successive radii *r_i_*_+ 1_ and *r_i_*, hence its area being given by:

(17) $$SAHZ_{i}≅\pi \left(r_{i+1}^{2}-r_{i}^{2}\right)$$

This specific area has a 3 pixel width and contains 162 pixels. The *i*-th order AHZ can be estimated using the structural constant *K_T_* by the following equation:

(18) $$SAHZ_{i}=\frac{162}{pxz_{i}.K_{T}}$$

where *psz_i_* is the value of pixel number in AHZ_i_ normed to *K_T_*.

In each AHZ consisting of 54 circular trapezoidal surfaces, 162 pixels can be arranged in order to satisfy the discrete pixel density curve function of the eccentricity for the required *K_T_* corresponding to that specific AHZ surface (Figure 2b).

Combining Equations (17) and (18) one obtains:

(19) $$r_{i+1}=\sqrt{\frac{162}{pxz_{i}K_{T}\pi }+r_{i}^{2}}$$

Now, using our initial radius $r_{1}=0.175\;$ mm as well as the pixel density from Figure 2b, $pxz_{1}=64$ and $K_{T}=32$ one obtains:

(20) $$r_{2}=\sqrt{\frac{162}{64\times 32\times 3.14}+0.175^{2}}=0.235\;\mathrm{mm}$$

The radii values for the next five AHZs are: *r*_3_ = 0.295 mm, *r*_4_ = 0.355 mm, *r*_5_ = 0.410 mm, *r*_6_ = 0.465 mm, and *r*_7_ = 0.520 mm. The entire lattice can be generated by Equation (19).

In Figure 6a, we generated a lattice obtained by the planar projection of the superposition of the 27 circular surfaces on the first six AHZs corresponding to the foveal avascular zone (FAZ), resulting a configuration of 162 circular trapezoids. Each circular trapezoid will contain 3 pixels, hence the total number of pixels in the above lattice is 486. Hence, the entire array represented only by half in Figure 6c counts, in total, 5832 pixels. This computation method, as we have just illustrated on the FAZ surface, can be extended further to the entire macula (5.5 mm) covered by approximately eight AHZs, i.e., a total of 6966 (~7000) pixels. 

Three types of geodesics obeying Equation (15) have been plotted on the circular trapezoid configurations satisfying the pixel density in Figure 2b, as well as the requirement that each pixel should be on a geodesics. The geodesic in Equation (15) has been numerically integrated on the interval 48.5° ≤ *θ* ≤ 76° (in steps of 0.5°), corresponding to the prosthetics radii in the range 0.175 mm ≤ *r* ≤ 0.520 mm, (i.e., the FAZ surface) and taking *c*_0_ = 0.223, for both positive and negative geodesics (red and green color curves in Figure 6b, respectively). The same integration parameters (but we have taken *c*_0_ = 0.127) have been used for the third geodesics (magenta color curve in Figure 5). The *c*_0_ = 0.223 geodesics connect the corners of circular trapezoids, while the *c*_0_ = 0.127 geodesics connect the centers of two trapezoids. Each half pseudosphere contains 27 geodesics of each type separated by an angle corresponding to the center angle of each circle sector. 

In order to project the 2 × 3 panel (Figure 5g) into a circular shape to obtain the configuration in Figure 6, we will take note of the biunivocal correspondence between the 2 × 3 panel columns and circle sectors (a hemipseudosphere has 27 circular sectors corresponding to 27 columns from a 2 × 3 panel). For optimal processing of the incident energy, the pixels must be situated on the geodesics’ surface [33,51]. 

## 3. Discussion 

An AHM based on the multi-level model presented in this work is suitable to be implemented in Alpha-IMS type systems. We argue in this section that this model significantly ameliorates visual function by the improvement of stereoscopy, light sensitivity, visual acuity and of the color and contrast perception. We also argue that these improvements are achievable by fulfilling some challenging technical specifications (such as acceptable implantable size and satisfactory processing speed [52]) or biocompatibility requirements (such as reduced power consumption and reduced local heat dissipation) as well as the customization of VPDs for specific patients.

In order to achieve optimal visual function, we stress the importance of the prosthesis shape allowing a retinal excitation close to the “natural” excitation in healthy subjects with important impact on excitation processing in the brain. A subretinal VPD implantation of this type would also maintain a hierarchical structure similar to that of the biological retina [52]. 

The modification of the rectangular equidistant electrode array in the current camera-free VPDs is the fundamental strategy in the personalization of the device upon the disease stage for each patient. There is a general consensus nowadays that the visual space has a hyperbolic geometry [53]. On the other hand, the neuronal area V_1_ is a hyperbolic planform in relation to texture perceptions [54]. Since the visual space and the V1 area can be described within the hyperbolic geometry, the stereoscopy is due to the common class of geometrical features of all the visual pathway elements: visual space, retinal surface and V1 area. Binocular depth perception depends on lateral disparity of the proximal stimuli on the retinas of both eyes [55]. In this way, human vision can perceive relief—it is stereoscopic. The stereoscopic input involves higher-order disparate image structures, not disparate retinal positions [56]. The two images from each eye are combined into a singular one in the V1 visual cortex [57]. The V1 area receives feed-back information regarding disparity from V2, V3 and the middle temporal areas, which have some cells that are sensitive to the difference in angles of the two retinas [58]. 

In the context of impaired retinas, in order to keep the stereoscopy, the prosthesis should share similar geometrical features. Our AHM concept based on this geometry aims to use the same already formed visual pathway by providing the input signals as close as possible to the patient’s natural input in order to encourage a natural processing of visual information in the brain. 

In addition to pixel orientation on the AHM surface, in order to further get closer to the aimed photoexcitation the pixel diameters and the distance between the pixels must be similar to that of the macular cone distribution. Within our model, the required distribution pixel-eccentricity is achievable through discreetly increasing the diameter of the pixel and the interval between the pixels, while keeping the pixel density coefficient *K_T_* constant (Figure 2b). In addition, the EPS for a single prosthetic canvas is larger than the area of the sphere sector of the same radius, and so it will contain the highest possible pixel number, and thus exhibits the greatest visual acuity. Hence, an EPS shaped AHM is ideal for achieving a pixel density distribution proportional to the natural distribution of the cones in the macula. This approach is supported by some small scale animal studies [59,60] and clinical [13,61,62,63] studies showing that patients with such types of prosthesis showed improvements in visual function, such as VA and perceptions of color, contrast and darkness.

A Snellen acuity of 20/20 represents normal vision, and 20/200 is defined as legally blind. The minimum visual acuity required for reading is 20/80, which implies pixels smaller than 20 μm, corresponding to 2500 pixels/mm^2^ in the center of the FV [64]. Retinal implants with the features described in this work are composed of a few thousand (~7000) photodiodes in the interest areas, each representing a pixel (Figure 7). The clinical results from retinal prostheses have been reviewed recently [65]. Nevertheless, the visual resolution obtained from existing devices is very limited, meaning that even recognizing simple objects is challenging. Until now, there is no rectangular shaped prosthesis satisfying the visual acuity requirement of 20/80 which would allow reading capital letters. The best acuities reported in the literature so far from clinical trials are 20/1260 from Argus II [66], 20/546 from Alpha-AMS [67] and between 20/4451 and 20/21,059 from BVA suprachoroidal devices [68], all within legal blindness. Crucial abilities, such as facial recognition, are not yet possible [69]; however, the proposed model is susceptible to lead to a solution for such a problem.

Moreover, the microsaccade amplitude is in relation to pixel size, i.e., the decrease in pixel diameter prevents undesired processes such as local adaptation and image fading [63]. In addition to a high pixel density, this strategy also allows the use of microsaccades for refreshing the perceived images [13]. This arrangement is also the most effective solution for the efficient sampling of signals transmitted to central nervous level [38]. The amount of color pixels is an important advance with respect to the current Alpha-IMS system [8,10,11,12], which is designed with 1600 pixels. 

It is known that a significant number of ganglionar cells are still viable at higher retinal eccentricities. The number of such functional cells still using the natural visual pathway and their distribution around the macula are unique for each patient. By VPD customization, we imply the prosthetic implantation to be superposed on the illness affected macular surface only. Such an approach, which of course has its difficulties, will have the advantage of a more rapid adaptation of the communication between the receptor area and the brain visual areas. However, such prosthetic adaptation has greater chances to be successful if the AHM surface follows the geometry described in Section 2.3 and the artificial cone cells (i.e., pixels or photodiodes) are distributed on such a surface as described in Section 2.4.

The AHM can be customized further at a smaller scale by the imitation of the cone cell distribution in healthy areas. The customization will consist of changing the elementary construct panels (c and/or d, for example) in Figure 5. Obtaining a cone cell sensitivity distribution in the prosthetic area closer to that of the healthy area has greater chances to improve color sensitivity. It has been suggested that the cone cell type distribution in the normal retina is explained by a random genetic process which mediates L versus M gene expression [49]. For example, measurements performed on retinas of various subjects at 1.25 ° nasal eccentricities lead to the following distributions of the three types of cone cells: 5.55% S, 27.77% M and 66.66% L (for a 1M→1L type mutation), 5.55% S, 22.22% M and 72.22% L (for a 2M→2L type mutation), and 5.55% S, 38.88% M and 55.55% L (for a 1L→1M mutation) [45]. The customization of the pixel distribution to achieve the variable ratios above on the EPS canvas can be generated automatically by a computer routine.

The acquisition of information about light polarization with the visual prosthesis would improve contrast perception by enabling the patients to recognize hard-to-distinguish differences between transparent surfaces and non-transparent surfaces, and to detect information on the surface orientation of single-colored objects. The phenomenon originates in the transmission of oblique light subject to Fresnel’s laws [70] in the specific S-type cone distribution of the fovea [71]. Prosthesis calibration based on the congruence of geodesics described by the S type cone cells should lead to a better probability for the focal activation of retinal ganglion cells.

In some of the previously mentioned studies [61,62], a major issue of subretinal prostheses was the lack of viable power sources, which do not transfer dangerously amounts of heat to the biological tissues. By matching the macular convexity, the EPS-shaped AHM allows a photodiode distribution which meets the requirement imposed on the distance between the electrodes and macular bipolar cell layer. This distance, which should be around 7 μm, maximizes the prosthetics’ efficiency and minimizes the electric power required for the prosthetic function [64]. Furthermore, the photodiode density decreases with the eccentricity, i.e., the distance to the FV. Based on the linear relation between the diameter and the eccentricity, the distance between the photodiodes increases by a factor of 1.4 to 2 [40]. Such variation favors the light-current conversion and the efficient absorption of the heat resulting from such conversion. 

In the visual prosthetic devices manufacture process, the most important challenge is the realization of the microelectrode array corresponding to the pixel density/eccentricity according to the discrete curve in Figure 2b. Without being exhaustive, we mention here two types of hybrid nanotechnology materials susceptible to satisfy the mathematically proven specifications for the photodiodes playing the role of pixels: organic photovoltaic (OPV) materials and vertically aligned carbon nanotubes (VACNTs) connected with quantum dots (QDs).

Organic technology is fast and easy to produce thanks to deposition methods such as ink-jet printing [72], which is easily adaptable to geometrical constrains up to a few square micrometers. The neural interfaces with organic semiconductors work without any externally applied electric field and with minimal heat dissipation [73]. Organic semiconductors may allow us to achieve the spectral sensitivities of natural color photoreceptors [74]. Such a technology has already been successfully applied in a fully organic device composed of a flexible and highly conformable silk substrate covered with photoactive layers of OPV conjugated polymers as interfaces for neuronal photostimulation and implanted in the subretinal space of dystrophic rats [75]. Furthermore, an optimal combination of bulk carrier concentration, diffusion length scales and the presence of different transport mechanisms prevalent in such structures could allow them to be used as single-pixel color sensing elements [14,17]. Organic photovoltaics [16,73] have a high biocompatibility potential, but light-current conversion is still low and 47% of energy turns into heat [76]. At a high density of pixels, it can lead to the destruction of organic tissue and becomes inoperable. Tandem systems represent a solution for the conversion of heat into electric energy so that the increase in light-induced temperature remains within the range of 1 °C, as stated in ocular safety regulations, Directive 90/385/EEC [77]. 

Neuronal photoactivation is possible using QDs in conjugation with vertically aligned carbon nanotubes (VACNTs), representing a cutting-edge technological solution. VACNTs have excellent electrochemical, biocompatible and durability properties [78], making them excellent candidates for ultradense microelectrode arrays (diameter 20–200 μm) [79,80]. VACNT-based electrodes deliver these stimulating pulses to the central nervous system optical fibers, initially targeting the surviving bipolar cells which transfer the signal through the ganglion cell layer. These microelectrodes are nearly purely capacitive [78], a required VPD property to avoid long-term operation tissue damage. QDs can absorb the excess thermal energy and then re-emit pure wavelength light through a mechanism of multiple excitons generation [81,82]. Through size control, QDs offer the possibility to engineer the band of visual cones via its spectral sensitivity for different wavelength ranges (red, blue, green) [81]. Some candidate QDs are made from materials with weak charge-phonon coupling, such as: lead sulfide (PbS), lead selenide (PbSe) and lead telluride (PbTe) [83]. Currently, the power conversion efficiency of VACNTs coupled with QDs is 10%, but in the future it is expected to exceed 33% [84]. 

## 4. Conclusions

In a bottom-up approach, in this paper we have proved several important geometric features of ocular structures and visual function in the framework of the non-Euclidian (hyperbolic) geometry. The apparent random distribution of cone cells has been modeled through a rigorous construct method in excellent agreement with the microscopic studies of retinal cellular structure. In the same framework, some structural features of the macula and of the ocular globe have been rigorously proved, allowing us to argue that superior visual function obeys similar hyperbolic projection laws. 

The highlighted finding of this paper, i.e., the photodiode array shape and high density pixel arrangement (based on our vision model) and the pixel design (based on the literature review) have been merged into a theoretical sub-retinal prosthetic device to be included in a camera-free visual prosthetic system. Our approach of human vision together with the prosthetic model might be a potential next step in the already-promising field of camera-free visual prosthetic systems, thanks to several features we discuss: color perception through individualized high pixel density (almost 7000 compared to the 1600 already available), polarized light perception, efficient power supply and thermal energy management, etc. Rigorous theoretical foundations for next generation microelectrode arrays have been laid out, inviting further reflections, implementation and testing. The manufacture of such an AHM will provide the possibility to deepen our understanding on the involvement of the differential HG in the determination of depth, movement and distance perception. 

## Figures and Tables

**Table 1:** Values of calculated complementary angles $A_{c},\;A_{c}^{\prime },\;A_{c}^{\prime \prime },\;A_{c}^{\prime \prime \prime }$ in the hyperbolic geometry Klein–Beltrami (HGKB) model and measured eccentricity angles corresponding to the human retina central region zones.

	Macula	Fovea	FAZ ^**^	Umbo
Calculated values of $A,\;A^{\prime },\;A^{\prime \prime },\;A^{\prime \prime \prime }$ ′ angles complementary to 60°	$A_{c}$	$A_{c}^{\prime }$	$A_{c}^{\prime \prime }$	$A_{c}^{\prime \prime \prime }$
	21.06°	5.88°	1.6°	0.36°
Values of the angular eccentricities *	18.20°	5.0°	1.4°	0.35°

* Measured values from [30] ** FAZ—foveal avascular zone.
